# Louisville Marine Hospital

**Published:** 1849-04

**Authors:** 


### LOUISVILLE MARINE HOSPITAL.

Courses of clinical instruction have been commenced at this institution, and will be continued through the vacation of the medical school. They will afford fine opportunities to students for acquiring a practical knowledge of medicine. Daily visits are made to the hospital, and students are present at the examination of patients by the physician and surgeon, hear the clinical remarks, and witness the post-mortem dissections.

Hitherto this excellent school for the study of practical medicine has been very much neglected by medical students during the summer recess, when, in fact, it is most valuable. It is in the vacation, when there is no crowd of students, that clinical medicine can be studied to most advantage. The courses now in progress at the hospital will commend themselves to all students of medicine who have it in their power to spend the spring and summer in the city. It is admitted by all, that the bedside is the place for attaining such knowledge of disease as fits the young physician for practice. Medicine, in all its departments, is demonstrative. The pupil, in order to understand, must see. Books will give him the elements; but he must make dissections, see cases, perform operations, institute diagnoses upon the sick, and acquire readiness in prescribing, before he is prepared to go forth as a practitioner. For these ends, hospitals are invaluable.

